# Supplementary material for: The interactome of intact mitochondria by cross-linking mass spectrometry provides evidence for coexisting respiratory supercomplexes
Source: Mol Cell Proteomics. 2017 Dec 8;17(2):216–32. doi: 10.1074/mcp.RA117.000470 (PMC5795388; doi:10.1074/mcp.RA117.000470)
Supplement: Supplemental Data [file supp_RA117.000470_133922_0_supp_23978_fzffwf.zip › spectra_annotation/mito_DR_spectra_annotation/96-1-1-1-12-1.pdf]

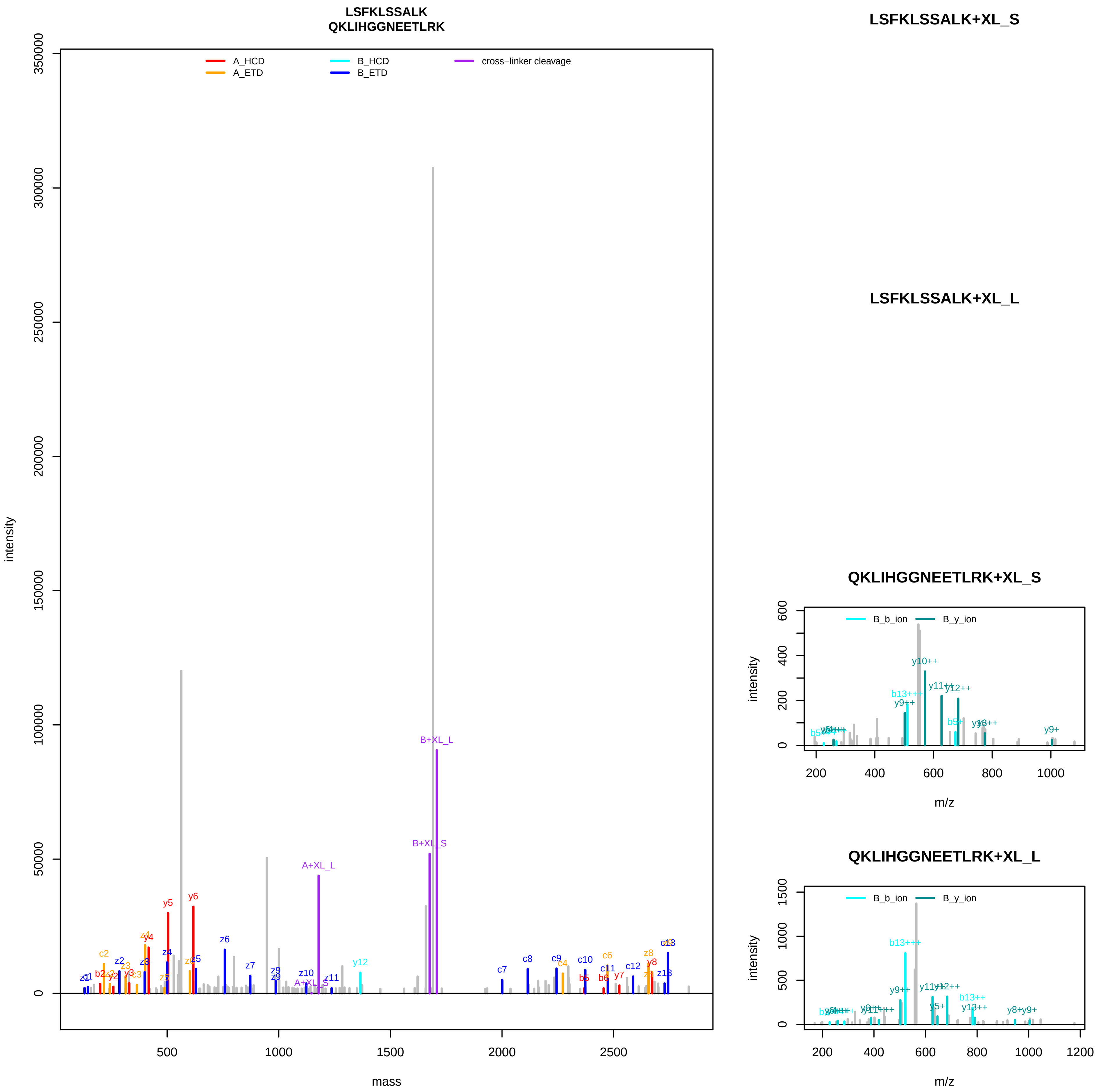

LSFKLSSALK
QKLIHGGNEETLRK
A_HCD
A_ETD
B_HCD
B_ETD
cross−linker cleavage
intensity
mass
0
50000
100000
150000
200000
250000
300000
350000
500
1000
1500
2000
2500
y5
y6
y4
z4
c2
z2
z3
z6
z7
z5
z9
z10
z11
y12
A+XL_L
A+XL_S
B+XL_L
B+XL_S
c7
c8
c9
c4
c10
c6
c11
c12
b5
b6
y7
z8
y8
c13
z13
LSFKLSSALK+XL_S
LSFKLSSALK+XL_L
QKLIHGGNEETLRK+XL_S
B_b_ion
B_y_ion
y10++
y11++
y12++
b13+++
y9++
y9+
m/z
200
400
600
800
1000
QKLIHGGNEETLRK+XL_L
b13+++
y9++
y11++
y12++
b13++
y13++
y5+
y8+y9+
1200
1500
